# Optimizing stroke prediction using gated recurrent unit and feature selection in Sub-Saharan Africa

**DOI:** 10.1016/j.clineuro.2025.108761

**Published:** 2025-01-27

**Authors:** Afeez A. Soladoye, David B. Olawade, Ibrahim A. Adeyanju, Onoja M. Akpa, Nicholas Aderinto, Mayowa O. Owolabi

**Affiliations:** a Department of Computer Engineering, Federal University, Oye, Ekiti, Nigeria; b Department of Allied and Public Health, School of Health, Sport and Bioscience, University of East London, London, United Kingdom; c Department of Research and Innovation, Medway NHS Foundation Trust, Gillingham ME7 5NY, United Kingdom; d Department of Public Health, York St John University, London, United Kingdom; e School of Health and Care Management, Arden University, Arden House, Middlemarch Park, Coventry, CV3 4FJ; f Department of Epidemiology and Medical Statistics, College of Medicine, University of Ibadan, Ibadan, Nigeria; g Division of Epidemiology, Biostatistics and Environmental Health, School of Public Health, University of Memphis, USA; h Department of Medicine and Surgery, Ladoke Akintola University of Technology, Ogbomoso, Nigeria; i Department of Medicine, University of Ibadan, Ibadan, Nigeria,; j Institute for Advanced Medical Research and Training, College of Medicine, University of Ibadan, Ibadan, Oyo, Nigeria; k University College Hospital, Ibadan, Nigeria

**Keywords:** Stroke prediction, Gated recurrent units, Machine learning, Feature selection, Medical diagnosis

## Abstract

**Background:**

Stroke remains a leading cause of death and disability worldwide, with African populations bearing a disproportionately high burden due to limited healthcare infrastructure. Early prediction and intervention are critical to reducing stroke outcomes. This study developed and evaluated a stroke prediction system using Gated Recurrent Units (GRU), a variant of Recurrent Neural Networks (RNN), leveraging the Afrocentric Stroke Investigative Research and Education Network (SIREN) dataset.

**Method:**

The study utilized secondary data from the SIREN dataset, comprising 4236 records with 29 phenotypes. Feature selection reduced these to 15 optimal phenotypes based on their significance to stroke occurrence. The GRU model, designed with 128 input neurons and four hidden layers (64, 32, 16, and 8 neurons), was trained and evaluated using 150 epochs, a batch size of 8, and metrics such as accuracy, AUC, and prediction time. Comparisons were made with traditional machine learning algorithms (Logistic Regression, SVM, KNN) and Long Short-Term Memory (LSTM) networks.

**Results:**

The GRU-based system achieved a performance accuracy of 77.48 %, an AUC of 0.84, and a prediction time of 0.43 seconds, outperforming all other models. Logistic Regression achieved 73.58 %, while LSTM reached 74.88 % but with a longer prediction time of 2.23 seconds. Feature selection significantly improved the model’s performance compared to using all 29 phenotypes.

**Conclusion:**

The GRU-based system demonstrated superior performance in stroke prediction, offering an efficient and scalable tool for healthcare. Future research should focus on integrating unstructured data, validating the model on diverse populations, and exploring hybrid architectures to enhance predictive accuracy.

## 1. Introduction

The integration of modern technological paradigms into the healthcare sector has significantly advanced medical science. Health plays a critical role in ensuring a productive and fulfilling life for individuals. Among the innovative technologies, Machine Learning (ML), a subset of Artificial Intelligence (AI), has emerged as a transformative tool in medicine [1]. Its application spans various medical processes, including disease prediction, diagnosis, surgical precision, and treatment, leading to more reliable, accurate, and faster outcomes [2–4]. Early prediction and diagnosis of diseases using ML are pivotal in developing treatment plans that can mitigate severe outcomes [5]. Diseases vary in their intensity and impact; while some may leave no lasting effects, others cause disabilities that render individuals vulnerable. Stroke is a prominent example of such a debilitating condition. Employing ML for early stroke prediction and diagnosis can enable timely interventions and precautionary measures to prevent or reduce its occurrence [6].

Stroke is a severe cardiovascular condition that leaves victims with lifelong disabilities, making it a medical emergency requiring prompt attention and prevention. Also known as Cerebrovascular Accident (CVA) or Cerebrovascular Insult (CVI), stroke occurs when blood flow to the brain is interrupted or blocked, cutting off its vital supply [7]. Developing countries face a disproportionately high stroke mortality rate, accounting for nearly 87 % of global cases, with Sub-Saharan Africa being a particularly affected region [8]. Factors such as inadequate technological advancements, limited medical resources, and negligence contribute to these statistics. Stroke remains a leading cause of death and disability globally. In the United States, approximately 795,000 individuals experience strokes annually [9].

Recognizing the severity of stroke, organizations such as the World Health Organization (WHO), American Heart Association (AHA), and World Stroke Organization (WSO) advocate for strategies to reduce stroke-related mortality, morbidity, and disabilities. These strategies focus on improving the accuracy and efficacy of stroke treatment methods, leveraging advanced technological tools, and raising awareness about risk factors. By identifying and managing these risk factors, potential stroke occurrences can be minimized [10]. This study aligns with these efforts by utilizing electronic medical records to analyze stroke-related phenotypes and predict its occurrence using a deep learning algorithm called Gated Recurrent Units (GRU).

Stroke is a prevalent and life-threatening condition that demands ongoing research to enhance prevention strategies. Numerous studies have explored methods for predicting stroke and raising awareness of associated phenotypes. For instance, a study employed electronic medical records from Okayama Prefecture, Japan, to predict stroke using GRU and CNN models [11]. The fused model demonstrated better performance with an Area Under the Curve (AUC) score of 0.669, though it lacked crucial risk factors like smoking and hypertension due to confidentiality constraints. Similarly, another used demographic and medical screening data to predict stroke, achieving superior results with an Artificial Neural Network (ANN) model [12]. Ahmed et al. implemented ML models, including Random Forest and Support Vector Machines, to predict stroke on an Apache Spark platform, with Random Forest yielding the highest performance [13].

Deep learning approaches have also been applied in stroke prediction. For example, Chen et al. used diffusion-weighted imaging data and a YOLOv5 algorithm to detect stroke lesions, achieving 79.86 % accuracy with a modified model [14]. Yang et al. and Lin et al. investigated classifiers like XGBoost and Random Forest on datasets from hypertensive patients and Taiwan’s stroke registry, respectively, highlighting their effectiveness in ischemic and hemorrhagic stroke prediction [15, 16]. Other researchers, such as Cheon, Kim, and Lim, leveraged deep neural networks for stroke prediction, while Zhao et al. combined traditional risk factors with multimodal retinal imaging for enhanced accuracy [17,18].

The rationale for this study stems from the critical need to improve early prediction and prevention of stroke, particularly in regions such as Sub-Saharan Africa, where the burden of stroke-related mortality and disability is disproportionately high due to limited technological advancements and healthcare infrastructure. While numerous studies have utilized machine learning and deep learning approaches for stroke prediction, the novelty of this research lies in its focus on leveraging a structured Afrocentric stroke dataset, which addresses the underrepresentation of African-specific data in existing models. Additionally, the study employs Gated Recurrent Units (GRU), a powerful yet underutilized deep learning algorithm, known for its ability to process sequential and temporal data effectively. The primary objectives of this study are threefold: first, to analyze electronic medical records (EMRs) to identify key phenotypes and risk factors associated with stroke; second, to develop and implement a GRU-based model for accurate stroke prediction; and third, to provide insights that can inform the development of tailored intervention strategies and healthcare policies aimed at reducing the incidence and impact of stroke in African populations. By addressing these gaps, this study not only advances the state-of-the-art in stroke prediction but also contributes to a more inclusive and effective global healthcare framework.

## 2. Methods

The proposed system comprises the following key components: Data Acquisition, Data Preprocessing, Feature Selection, Prediction, and Evaluation. Each component plays a critical role in ensuring the reliability and efficiency of the stroke prediction process. Data Acquisition involves collecting the necessary dataset, which forms the foundation for the entire prediction framework. Once acquired, the Data Preprocessing step is performed to clean and prepare the data by addressing inconsistencies, handling missing values, and normalizing the data to ensure compatibility with machine learning models. Following this, Feature Selection technique was applied to identify the most relevant features, reducing dimensionality and improving the computation time and model’s performance. The processed dataset is then divided into Training and Testing Sets, with the training set utilized for model training and the testing set reserved for evaluating the model’s performance.

**Fig. 1.**
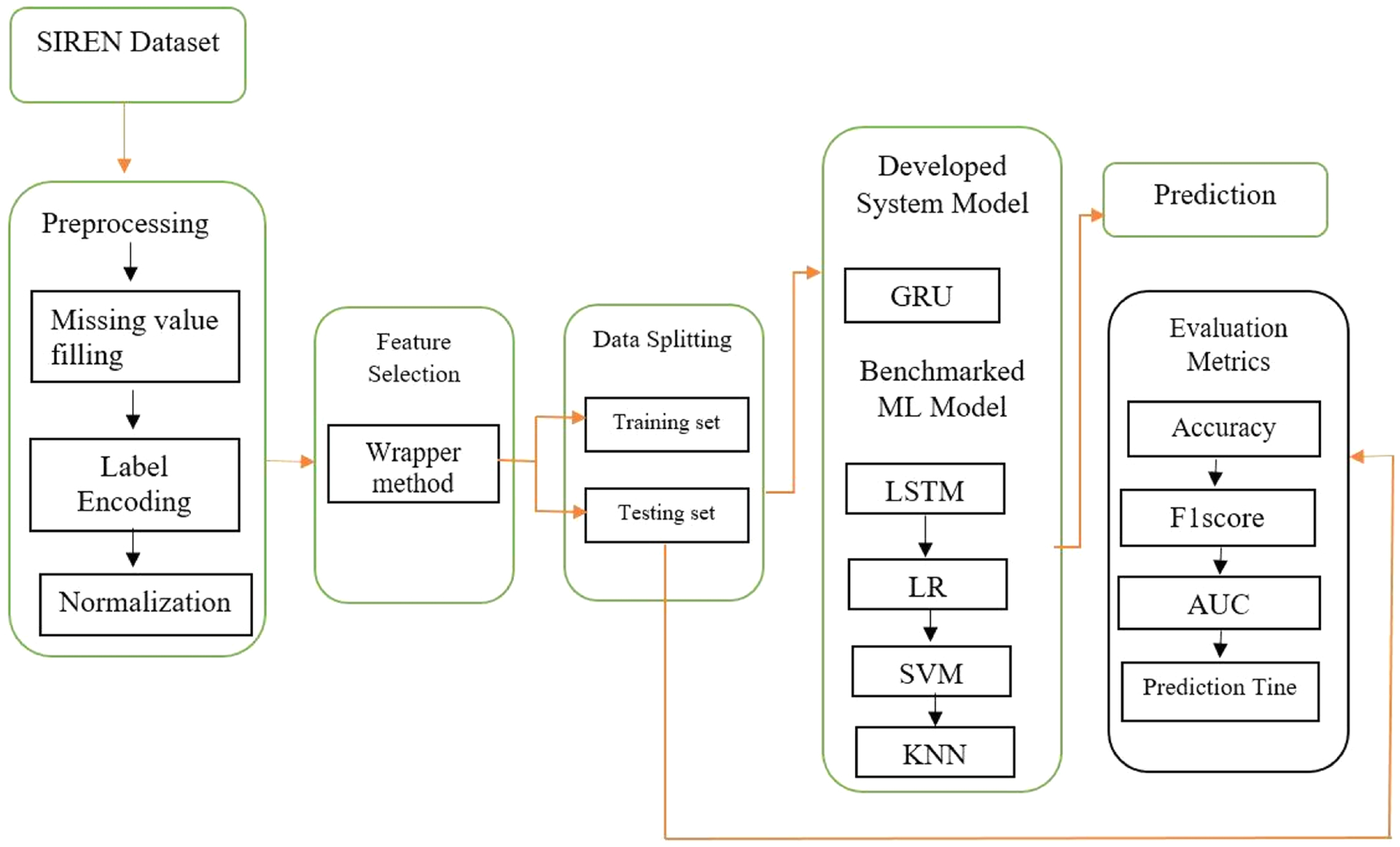
Block Diagram for Prediction of Stroke using GRU.

Fig. 1 illustrates the workflow of the proposed system, starting from the dataset acquisition through preprocessing and feature selection, and leading to model training and evaluation. The trained model is subsequently tested on the unseen testing dataset to validate its predictive accuracy. To ensure the robustness of the developed system, various machine learning algorithms were benchmarked against the proposed GRU-based model. Performance comparisons were conducted using evaluation metrics such as Accuracy, Area Under the Curve (AUC), Mean Absolute Error (MAE), F1-Score, and Prediction Time.

### 2.1. Data acquisition

The Afrocentric stroke dataset used in this study was obtained from the Stroke Investigative Research and Education Network (SIREN). Data collection involved collaboration with eight (8) centers across Nigeria and Ghana [19]. A total of twenty-nine (29) phenotypes were extracted from the dataset’s data dictionary based on insights from the reviewed literature. These phenotypes included a variety of demographic, lifestyle, and clinical factors such as Age, Gender, Smoking and Drinking Habits, History of Hard Drug and Sedative Use, Sleeping Disorders, Dietary Habits (Fruits and Vegetables Consumption), Physical Activity Levels, Family History of Stroke or Heart Attack, Blood Pressure (Systolic and Diastolic), Body Mass Index (BMI), Irregular Heartbeat, Atrial Fibrillation, Memory Impairment, History of Ischemic Stroke, Emotional Stress or Depression, Pulse, Migraine, Hypertension, Marital Status, Occupation, and Residence Type. The dataset comprised 4236 patient records, each containing information on these 29 phenotypes.

### 2.2. Data preprocessing

As with most medical datasets, the Afrocentric stroke dataset contained missing values. To address these, the median values of the respective phenotype columns were used to fill the gaps, ensuring robustness while mitigating the influence of outliers. For categorical data, a label encoder was applied to convert object values into integer format, since these were inherently numerical.

Certain phenotypes, such as Age, Systolic Blood Pressure (SBP), Diastolic Blood Pressure (DBP), and BMI, had significantly larger numerical ranges compared to binary phenotypes. To ensure even distribution and prevent these larger values from dominating the machine learning algorithms, these columns were normalized using the MinMax Normalization technique, as employed by Al-Shammari et al. [20]. The normalization process, represented in Eq. 1, transforms the values into a range between 0 and 1, enhancing the dataset’s suitability for predictive modeling.

(1) $$\text{x}=\frac{X-X_{\text{min}}}{X_{\text{max}}-X_{\text{min}}}$$

### 2.3. Feature selection

The researchers requested 29 phenotypes from the SIREN dataset, selected based on insights from the reviewed literature. However, these phenotypes required further analysis to identify the most significant ones contributing to the occurrence of stroke. To achieve this, a sequential forward-backward feature selection technique was employed to determine the optimal subset of phenotypes with the highest predictive significance. The analysis revealed that the following 15 phenotypes provided the best performance with the system: BMI, History of Hypertension, Cardiovascular Diseases, Diabetes, Depression, Blood Pressure, Atrial Fibrillation, Salt Consumption Habits, Vegetable Consumption, Education Level, Depression, Family History of Cardiovascular Diseases, and Stress. These selected phenotypes outperformed the others in contributing to the accuracy and reliability of the stroke prediction system.

### 2.4. Prediction of stroke using Gated Recurrent Unit (GRU)

Machine learning has been extensively employed for the prediction of various medical diseases due to its ability to provide faster, easier, and more accurate results. For every prediction task, once the dataset is cleaned, it is subsequently fed into the desired model for analysis and prediction. In this research, Gated Recurrent Units (GRU), a variant of Recurrent Neural Networks (RNN), was chosen as the primary predictive model. This choice was made due to GRU’s superior speed and performance compared to Long Short-Term Memory (LSTM) networks. Additionally, GRU has been less explored for structured datasets, as it is typically applied to streaming and time-series data, making this research a novel application of GRU.

What distinguishes RNNs from other deep learning algorithms is their ability to retain information through memory, making them particularly effective for sequential data. However, a key limitation of RNNs is their tendency to forget information over time, a challenge known as gradient vanishing. To address this issue, GRU and LSTM models were developed, incorporating specialized cells and gates to retain and process relevant information more effectively.

GRU simplifies the memory mechanism of LSTM by using just three gates and does not require internal cells to retain information. Instead, any information that needs to be preserved is integrated directly into the hidden state. The three gates of GRU are as follows:

- Update Gate (z): Determines the extent to which previously acquired knowledge from past inputs should be retained and forwarded to future states.
- Reset Gate (r): Identifies which parts of past information should be forgotten or omitted.
- Current Memory Gate (ht): Often overlooked by researchers, this gate is embedded with the reset gate and is responsible for storing the current information. It is also referred to as the hidden state, where information is stored, replacing the role of internal cells found in LSTM.

**Fig. 2.**
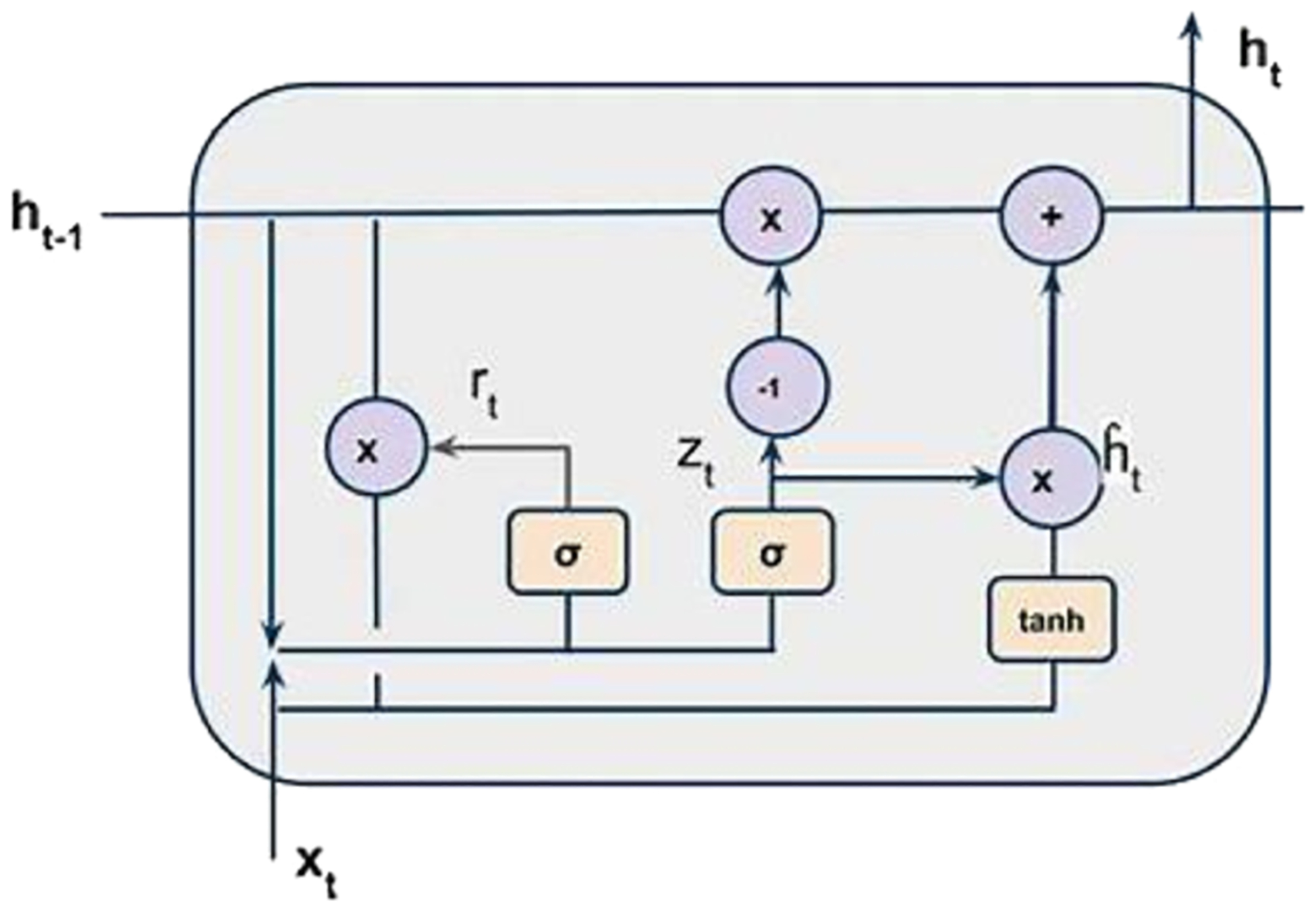
Overview of Gated Recurrent Unit (Chung *et al*., 2014).

Fig. 2 illustrates these GRU components, providing a visual representation for better comprehension of how the gates operate and interact. By employing GRU in this study, the system leverages its ability to effectively process structured data and mitigate gradient vanishing, thereby enhancing the accuracy and reliability of stroke prediction.

**Algorithm 1.** shows the algorithmic execution of the developed system for the prediction of stroke using Gated recurrent units and the evaluation using classification report.

**Algorithm 1.** RNN-GRU for Prediction of stroke with structured data

Step 1:Split the SIREN dataset (Sd) into training and testing sets

$${\text{Sd}}_{\text{Te}}=\text{Sd}\times 0.8$$

$${\text{Sd}}_{\text{Tr}}=\text{Sd}\times 0.2$$

Step 2:Convert the label testing set to Binary vector matrix using Utils.np

Step 3:Build the GRU models with input units.

Step 4:Initialize all the needed parameters matrices like ${\text{W}}_{\text{r}},{\text{W}}_{\text{z}},{\text{U}}_{\text{r}},{\text{U}}_{\text{z}},{\text{b}}_{\text{z}},{\text{b}}_{\text{h}}$, among others

For I ← to n do

Compute $z_{\left(t\right)}=\sigma_{g}\left(W_{z}X_{\left(t\right)}+U_{z}h_{t-1}+b_{z}\right)$

Compute $r=\sigma_{g}\left(W_{r}X_{\left(t\right)}+U_{r}h_{\left(t\right)}+b_{r}\right)$

Compute ${\ddot{\text{h}}}_{t}=\phi \left(W_{h}X_{\left(t\right)}+U_{h}\left(r_{\left(t\right)}⊙h_{\left(t-1\right)}+b_{h}\right.\right.$

Compute $h_{t}=\left(1-z_{t}\right)⊙h_{\left(t-1\right)}+z_{\left(t\right)}⊙{\ddot{\text{h}}}_{\left(t\right)}$

End for;

Step 5:Assign appropriate output based on the value of the target label

Dense (2, activation function)

Step 6:Compile the model applying activation function, loss (categorical_crossentropy) and optimizer

Activation function = Softmax (σ(z)i)

$$\sigma (\text{z})\text{i}=\frac{e^{zi}}{\sum_{j=1}^{k}e^{zj}}$$

Adam Optimizer

$${\text{M}}_{\text{t}}=\beta_{1}xM_{t-1}+\left(1-\beta_{1}\right)g_{t}$$

$${\text{V}}_{\text{t}}=\beta_{2}xV_{t-1}+\left(1-\beta_{2}\right){g_{t}}^{2}$$

$${\text{W}}_{t+1}=W_{t+1}=w_{t}-\frac{\eta }{\sqrt{V_{t}+\epsilon }}m_{t}$$

Step 7:Train the model using Epoch= 150, val_split = 0.2, Batch_Size= 64

Step 8: Predict with x_test.

Step 9:Print Classification report and Accuracy score.

Step 10: Evaluate with x_test and y_test

**Fig. 3.**
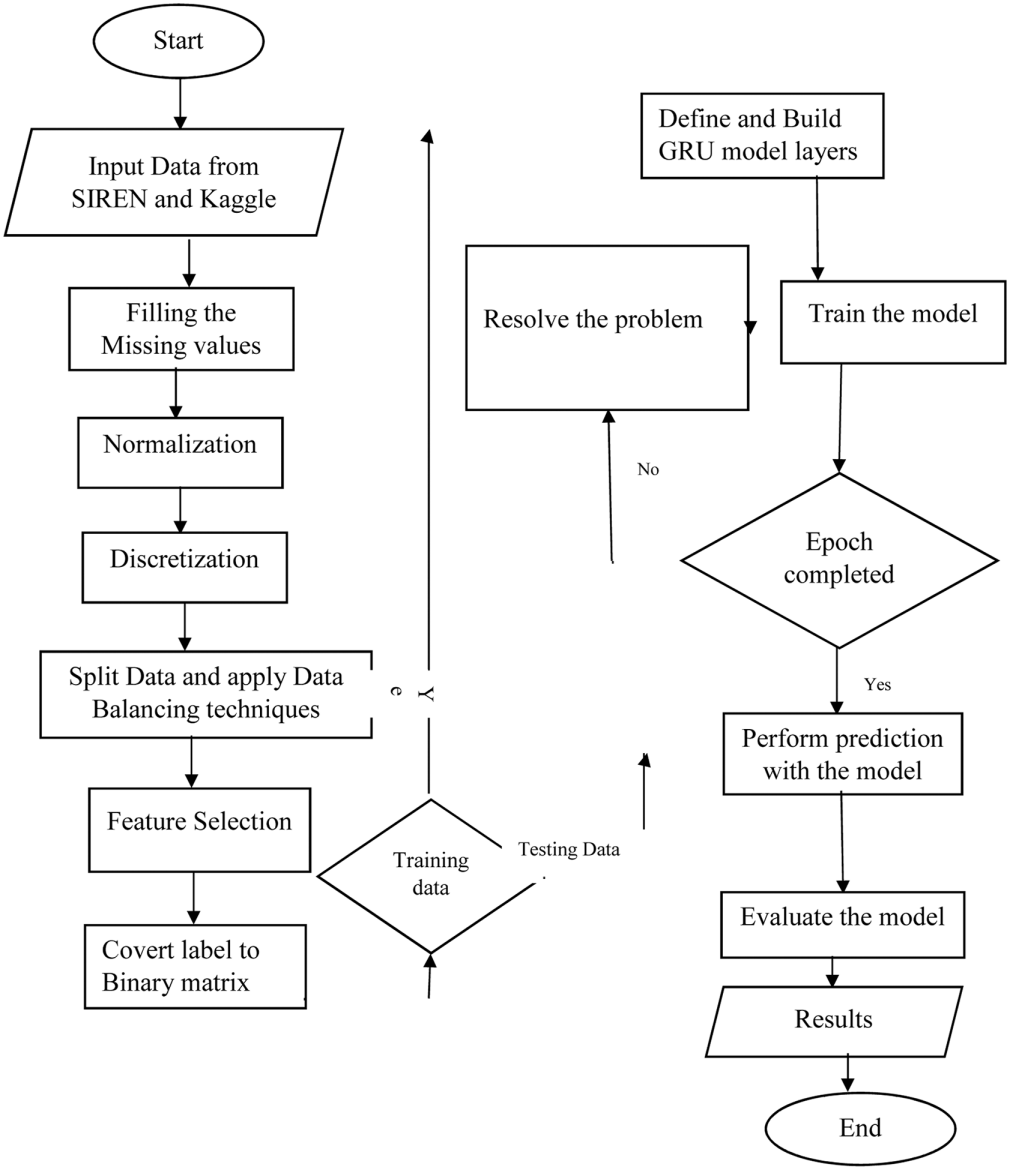
Flowchart of the Developed Stroke Prediction System.

Fig. 3 illustrates the flowchart of the developed stroke prediction system, detailing its key components and processes. The system begins with data acquisition from the Afrocentric SIREN dataset, followed by data preprocessing, where missing values are imputed and normalization is applied. Next, feature selection identifies the most relevant 15 phenotypes for model training. The preprocessed data is split into training and testing datasets, with the training set used to train the GRU-based model configured with optimized parameters. Finally, the system performs prediction and evaluation using metrics such as accuracy, AUC, and computation time. This flowchart provides a clear overview of the systematic approach used to develop and implement the predictive model.

### 2.5. System development and implementation

This study developed a system for stroke prediction using Python 3.9, implemented on Google Colab, alongside a local machine setup. A series of Python libraries, including NumPy, Pandas, TensorFlow, Scikit-learn, and Matplotlib, were employed to handle data preprocessing, model development, and visualization.

The GRU model was executed in two environments:

1. Local Machine Setup: A PC running Windows 10, equipped with 6 GB RAM and an Intel Celeron CPU.
2. Google Colab Virtual Machine: This environment provided a RAM of 12.68 GB and a disk space of 107.72 GB, enabling efficient execution of deep learning models.

The dataset used in this research was obtained as secondary data from the Stroke Investigative Research and Education Network (SIREN). It consisted of 4236 records, encompassing 29 requested phenotypes, making it a comprehensive resource for stroke prediction modeling.

### 2.6. GRU model architecture

The GRU model was designed with an input layer of 128 neurons and four hidden layers comprising 64, 32, 16, and 8 neurons, respectively. This architecture resulted in a total of 100,002 trainable parameters. Key configurations of the model included:

- Use of Bias and Return Sequence: Both set to True.
- Activation Functions:
  - Tanh for the input and hidden layers, ensuring non-linearity.
  - Softmax for the dense activation layer, used for multi-class classifcation but was employed for binary classification in this study.
- Regularization: A dropout rate of 0.2 was applied to reduce overfitting.
- Loss Function: The model was compiled with categorical_crossentropy, suitable for multi-class classification problems but used for binary classification.
- Optimizer: The Adam optimizer, known for its efficiency in deep learning applications, was utilized to minimize the loss.

This configuration enabled the GRU model to process the structured dataset effectively, leveraging its sequential processing capabilities for accurate stroke prediction. The system’s implementation highlights a balance between computational resource constraints and model complexity, ensuring optimal performance across both local and virtual environments.

### 2.7. Evaluation method and metrics

The hold-out evaluation method was employed in this study, with 20 % of the entire dataset set aside as the testing dataset. This approach ensured that the model was evaluated on unseen data, providing an unbiased estimate of its performance. To assess the performance of the developed system, several evaluation metrics were utilized, including accuracy, F1 score, Area Under the ROC Curve (AUC), Mean Absolute Error (MAE), and computation time.

Among these metrics, the AUC is particularly significant as it measures a binary classifier’s ability to correctly distinguish between classes. A higher AUC value indicates better model performance in separating positive and negative cases. The Mean Absolute Error (MAE) was also employed to quantify the average absolute difference between predicted and actual values, providing a direct measure of the model’s prediction accuracy. Additionally, computation time was evaluated to assess the model’s efficiency in making predictions.

Some of these evaluation metrics are mathematically represented in Eqs. 2 and 3, offering a formal framework for their computation and interpretation. These metrics collectively ensured a comprehensive assessment of the system’s performance, focusing on both its predictive accuracy and computational efficiency.

(2) $$\text{Accuracy}=\frac{TP+TN}{\left(TP+FP+TN+FN\right)}$$

(3) $$\text{F}1\text{score}=\frac{2*\text{precision}*\text{recall}}{\text{precision}\;+\;\text{recall}}$$

## 3. Results

The developed GRU-based system was tested with varying parameters to assess its performance under different configurations. Additionally, comparisons were made with other machine learning algorithms and existing systems to identify the best-performing approach. Consistently, a validation split and a dropout rate of 0.2 were employed throughout the experiments. This section provides an in-depth analysis of the experimental results and highlights the impact of parameter variations.

### 3.1. Empirical results with varying GRU parameters

To determine the optimal configuration for the GRU model, experiments were conducted using different combinations of input layers, hidden layers, epochs, and batch sizes. The testing began with small input and hidden layers, progressively increasing the number of neurons to evaluate their influence on the system’s accuracy. The results, as summarized in Tables 3 and 4, compare the performance when using all 29 phenotypes with the performance with when using only the 15 optimal phenotypes identified earlier.

As presented in Table 3, when all 29 requested phenotypes were used as inputs, the system achieved its highest average performance accuracy of 72.88 %. This was attained with a configuration that included 64 neurons in the input layer, two hidden layers with 32 and 16 neurons, respectively, 50 epochs, and a batch size of 16. While other configurations, such as those yielding 72.76 % and 72.52 % average accuracy, came close, they did not outperform the optimal setup.

The findings demonstrate that adjusting parameters, such as the number of neurons, batch size, and epochs, can significantly affect the system’s performance. However, beyond certain thresholds, improvements diminish, highlighting the importance of balancing model complexity with computational efficiency. These insights emphasize the adaptability of the GRU model to structured datasets, providing a strong foundation for accurate and efficient stroke prediction.

As shown in Table 4, when only 15 phenotypes were used, the developed system achieved the highest average performance accuracy of 77.48 %, with a prediction time of 0.42 seconds. This configuration outperformed other setups, demonstrating the significance of selecting optimal phenotypes for improved performance. The closest accuracy to this was 76.65 %, obtained from a system designed with an input layer of 12 neurons and hidden layers comprising 64, 32, 16, and 8 neurons, with 100 epochs and a batch size of 32. This system had a slightly longer prediction time of 0.43 seconds.

The results presented in Table 4 highlight that the system using only 15 phenotypes consistently outperformed those using all 29 phenotypes. This demonstrates the importance of effective feature selection, as reducing the dataset to the most relevant phenotypes not only improved performance accuracy but also resulted in comparable or faster prediction times. These findings underscore the efficiency of the developed system and its ability to achieve superior performance with fewer, well-selected phenotypes.

### 3.2. 3.2 Experimental result of the developed system

The best performance in this study was achieved using the preprocessed SIREN dataset and a GRU model configured with 128 neurons in the input layer and four hidden layers comprising 64, 32, 16, and 8 neurons, respectively. This design delivered the highest performance metrics when utilizing 15 phenotypes out of the 29 initially requested. These selected phenotypes included BMI, history of hypertension, cardiovascular diseases, diabetes, depression, blood pressure, atrial fibrillation, salt habit, vegetable consumption, education, family history of cardiovascular diseases, and stress. The model achieved an accuracy of 77.48 %, an AUC of 0.84, and a prediction time of 0.42 seconds.

The performance evaluation, as presented in Table 4, highlights the significance of the selected phenotypes and the effectiveness of the GRU model configuration. By leveraging the most impactful phenotypes and a well-optimized deep learning architecture, the developed system demonstrated superior performance compared to configurations using all 29 phenotypes.

### 3.3. Training and validation performance

**Fig. 4.**
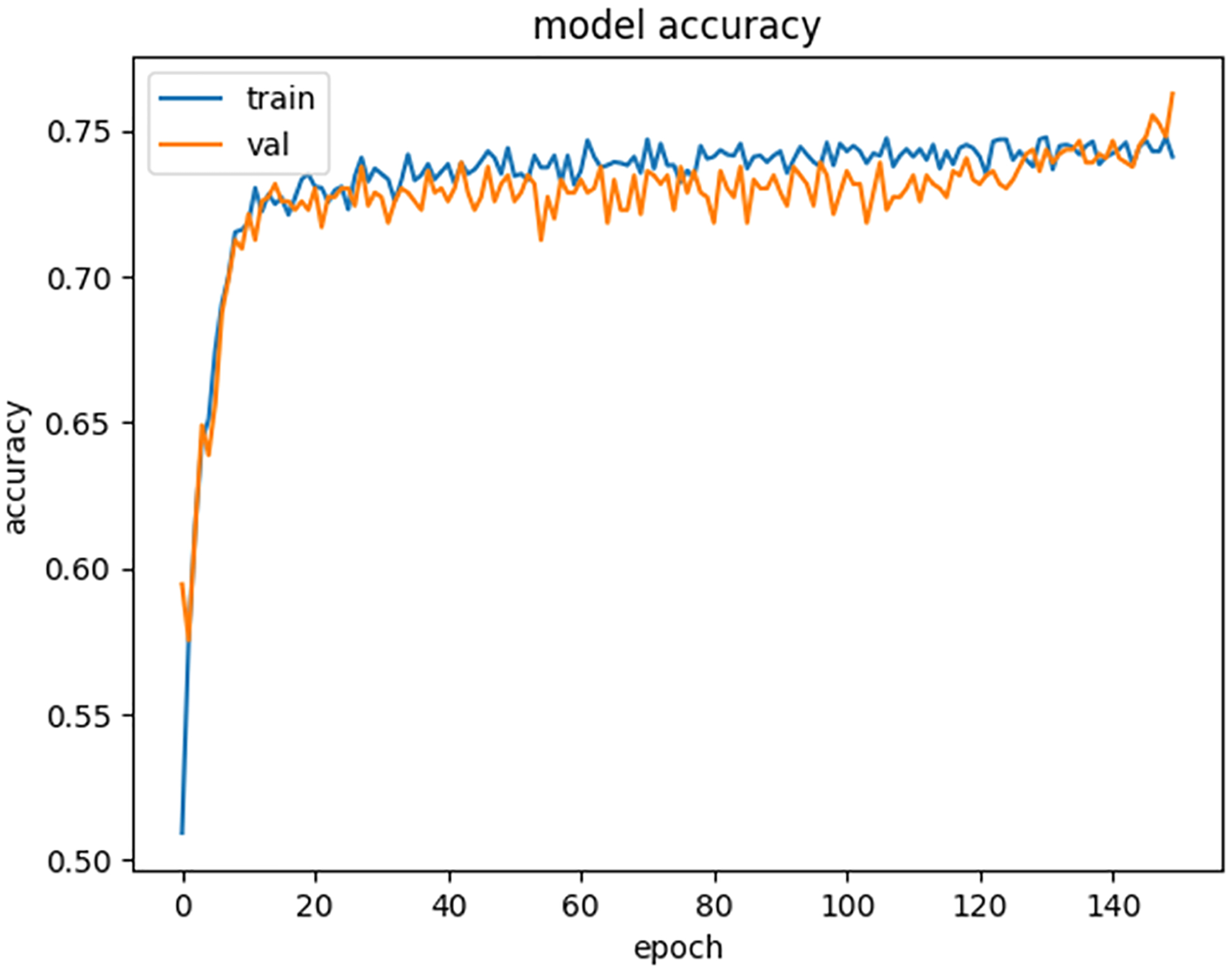
Training and Validation accuracy graph.

Fig. 4 illustrates the training and validation accuracies achieved during the training of the developed system, which was configured with 150 epochs and a batch size of 8. The model’s training accuracy exhibited a consistent range between 73 % and 74 % across epoch values from 20 to 150, indicating that the optimal performance was achieved as early as the 20th epoch. Meanwhile, the validation accuracy fluctuated between 71 % and 72 % from epochs 20–100 and significantly improved between epochs 100 and 150, reaching a maximum value of 76 %.

Throughout the training process, the training accuracy slightly exceeded the validation accuracy, except during the final five epochs, where the validation accuracy surpassed the training accuracy. This behavior implies that the model avoided overfitting or underfitting, demonstrating effective generalization and consistent performance across the dataset.

**Fig. 5.**
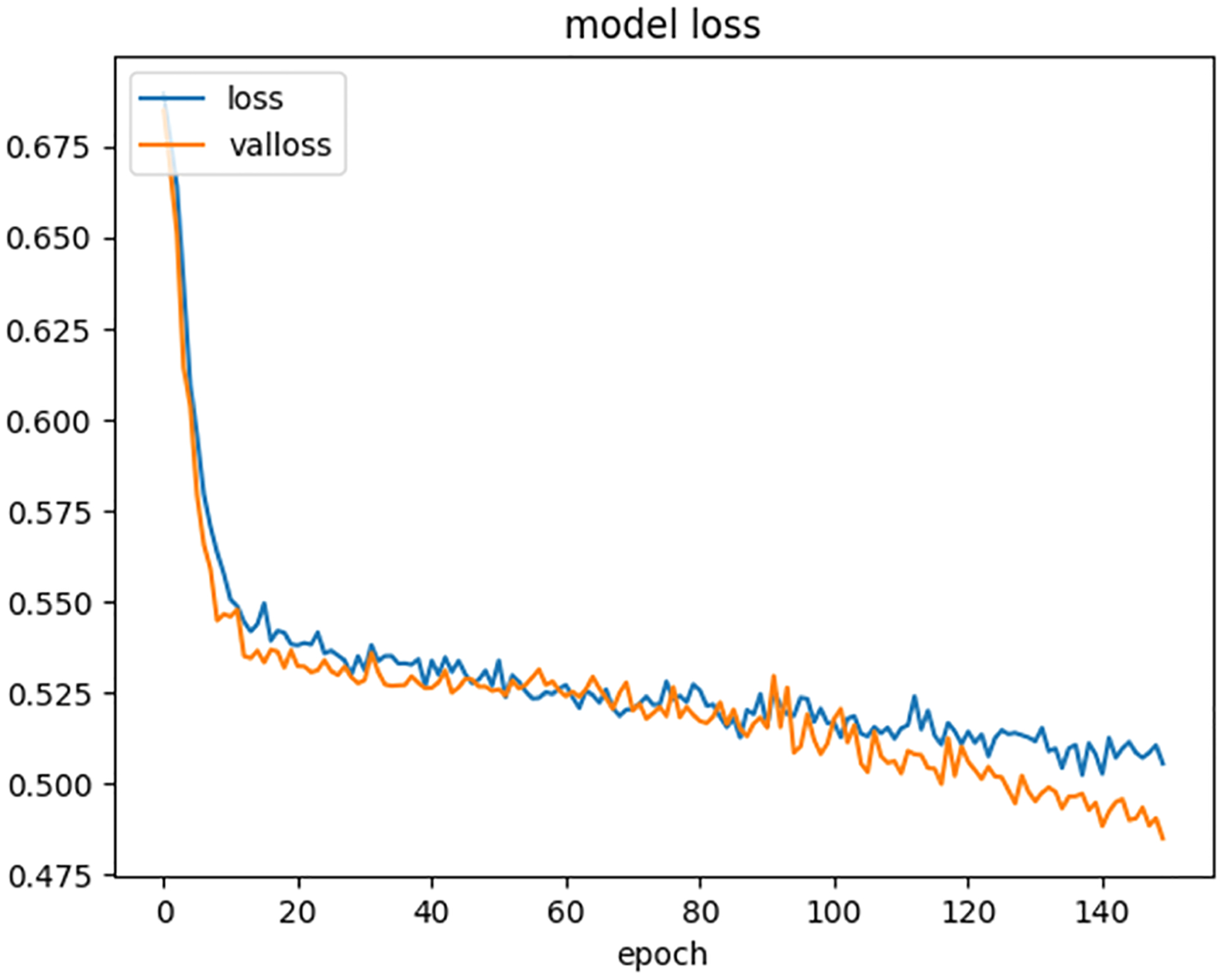
Training and Validation Loss Graph.

Fig. 5 depicts the training and validation losses recorded during the model’s training. As expected, both training and validation losses decreased progressively, indicating that the model was learning effectively. Between epochs 100 and 150, the validation loss dropped slightly below the training loss, signifying that the validation split of the dataset contributed to better performance and provided a lower validation loss value.

These observations collectively highlight the robustness of the developed system, its ability to generalize well across training and validation datasets, and its consistent performance without overfitting or underfitting during training.

As shown in Table 5, the developed system demonstrated a robust performance, achieving an average accuracy of 77.48 %, a prediction time of 0.42 seconds, and an evaluation time of 0.071 seconds. These results highlight the system’s efficiency, particularly in terms of speed, as the time required to perform a single test is remarkably low at just 0.071 seconds. Furthermore, the system recorded a Mean Absolute Error (MAE) of 0.31, indicating a high level of precision in its predictions. This combination of accuracy, speed, and minimal error underscores the effectiveness and practicality of the developed system for stroke prediction, making it a reliable tool for real-time and large-scale applications.

### 3.4. Comparison of the developed system with some machine learning algorithm

The developed system utilized Gated Recurrent Units (GRU), a variant of Recurrent Neural Networks (RNN), as the primary prediction algorithm. GRU distinguishes itself from other neural networks due to its ability to incorporate memory, enabling the retention of relevant information over time. This feature makes GRU particularly suitable for sequential data and prediction tasks.

In this study, the performance of GRU was compared with several commonly used machine learning algorithms, including Support Vector Machine (SVM), K-Nearest Neighbor (KNN), Logistic Regression (LR), and Long Short-Term Memory (LSTM), which is another widely adopted RNN variant. The comparison followed the same methodology, using identical datasets and evaluation metrics to ensure a fair assessment.

Given that LSTM, like GRU, is a variant of RNN, it was specifically compared in detail, using varying parameters to analyze their respective performance. The results of this comparison, including accuracy and prediction time as the evaluation metrics, are presented in Table 6. These results provide insights into the relative efficiency of GRU compared to LSTM and other traditional algorithms, emphasizing the advantages of GRU in terms of accuracy and computational speed.

As presented in Table 6, while both LSTM and GRU layers were evaluated, they were not built with identical configurations of input and hidden neurons. This discrepancy arises from the inherent structural differences between the two models. LSTM, having a greater number of gates compared to GRU, generates more parameters when designed with the same number of neurons. Consequently, this impacts its computational efficiency and model complexity.

From Table 6, the GRU-based system outperformed LSTM in terms of both accuracy and prediction time. The GRU achieved the highest performance accuracy of 77.48 % with a prediction time of 0.43 seconds, whereas the LSTM recorded a lower accuracy of 74.88 % and a significantly longer prediction time of 2.23 seconds.

As shown in Table 7, a comparison was made between the developed system, built with Gated Recurrent Units (GRU), and traditional machine learning algorithms such as Logistic Regression (LR), Support Vector Machine (SVM), and K-Nearest Neighbors (KNN) with k = 3. Among these models, the developed GRU-based system achieved the highest performance accuracy of 77.48 %, outperforming all the traditional algorithms. Logistic Regression was the closest competitor, achieving an accuracy of 73.58 %, while KNN (k = 3) recorded the lowest accuracy at 71.82 %.

## 4. Discussion

This study successfully developed and evaluated a stroke prediction system using Gated Recurrent Units (GRU), a variant of Recurrent Neural Networks (RNN), and benchmarked its performance against traditional machine learning algorithms and Long Short-Term Memory (LSTM) networks. The results underscore GRU’s potential as a robust and computationally efficient model for stroke prediction, particularly when paired with optimal feature selection. The GRU-based system achieved an accuracy of 77.48 %, with a prediction time of 0.43 seconds, outperforming Logistic Regression (73.58 %), K-Nearest Neighbors (71.82 %), and LSTM (74.88 %), which also demonstrated significantly longer prediction times of 2.23 seconds. These findings highlight the key advantages of GRU, including its improved performance, faster inference, and suitability for real-time applications, making it a promising tool for stroke prediction in resource-limited settings.

The findings of this study align with existing evidence in the domain of machine learning for medical prediction. GRU’s ability to handle sequential data with fewer gates and parameters compared to LSTM makes it not only computationally efficient but also well-suited for structured datasets. Studies have highlighted GRU’s advantage in balancing computational demands with performance, which is particularly important for real-time applications where speed is crucial [21,22]. The efficiency of GRU observed in this study is consistent with evidence from prior research suggesting that RNN variants, including GRU, perform well in tasks requiring temporal or sequential data analysis, such as stroke prediction, which involves patterns over time that are crucial for accurate outcomes.

An important aspect of this study was the role of feature selection in improving model performance. By reducing the original 29 phenotypes to 15 optimal ones, the system achieved higher accuracy and faster prediction times. This result resonates with the findings of Shivani et al., who demonstrated that reducing noise in datasets through feature selection enhances machine learning model performance by enabling the model to focus on the most relevant variables [23]. Similarly, Hassan et al. observed that employing feature selection techniques in time-series algorithms improved prediction accuracy for stroke risk factors [24]. The ability of GRU to deliver high accuracy with a reduced feature set suggests its practical applicability in environments where computational resources may be limited. This makes the GRU-based system not only accurate but also efficient and scalable, which is critical in low-resource settings such as Sub-Saharan Africa, where healthcare infrastructure may be challenged by limited computational capacity.

LSTM has been a popular choice in predictive modeling for medical applications due to its ability to handle long-term dependencies in sequential data. However, this study found that GRU consistently outperformed LSTM in terms of both accuracy and computational efficiency. While LSTM achieved an accuracy of 74.88 %, with a prediction time of 2.23 seconds, GRU achieved 77.48 % accuracy with a significantly faster prediction time of 0.43 seconds. These results highlight GRU’s efficiency, particularly in structured datasets where LSTM’s additional gates, while useful in time-series data, do not significantly enhance performance but instead increase computational costs. This observation is in line with conclusions drawn by Tu et al., where GRU demonstrated superior performance in specific prediction tasks, particularly when processing datasets with minimal temporal complexity [25]. The ability of GRU to provide similar or better predictive accuracy with less computational overhead makes it a more suitable choice for stroke prediction in real-world healthcare applications.

In comparison to traditional machine learning algorithms, the GRU-based system also demonstrated superior performance. Logistic Regression, which achieved 73.58 % accuracy, was the closest competitor to GRU. However, its reliance on linear assumptions and inability to capture complex, non-linear relationships within the data limited its predictive power. K-Nearest Neighbors (KNN) and Support Vector Machine (SVM) achieved even lower accuracies, with KNN (k = 3) producing an accuracy of 71.82 %, highlighting the limitations of traditional algorithms in handling multidimensional and complex datasets. These results align with prior research, which noted the limitations of traditional algorithms when applied to high-dimensional medical datasets [26]. Traditional algorithms such as Logistic Regression, KNN, and SVM struggle to capture complex interactions between variables, which is essential for accurate stroke prediction. In contrast, deep learning models like GRU excel at capturing non-linear relationships, making them more suitable for complex, high-dimensional medical data.

The results of this study have significant implications for the development of stroke prediction systems, particularly in resource-limited settings like Sub-Saharan Africa. The GRU-based model’s combination of high accuracy and fast prediction time makes it a promising tool for real-time stroke prediction, which is critical for timely intervention and improving patient outcomes. The integration of machine learning into stroke care could enable early identification of individuals at high risk, allowing for preventive measures or more focused monitoring to be implemented. Moreover, the ability to work with fewer features without sacrificing accuracy is particularly beneficial in regions where data collection may be incomplete or where the computational infrastructure is not robust enough to support more complex models.

Future research should aim to refine and validate the GRU-based model in a broader range of clinical settings, using diverse datasets that capture a variety of demographic, environmental, and clinical factors. While this study focused on a specific set of phenotypes, additional research exploring the inclusion of other variables, such as lifestyle factors, environmental exposures, or genetic predispositions, could further enhance prediction accuracy. Moreover, future studies could investigate the integration of multimodal data, combining clinical records, imaging data, and even wearable health data, to improve the predictive power of the model. Another avenue for future research could involve exploring the integration of the GRU-based stroke prediction system into clinical decision support tools, enabling healthcare providers to make data-driven, real-time decisions that could potentially save lives.

## 5. Strengths of the study

This study demonstrated significant contributions to the field of stroke prediction, particularly in underrepresented regions. By leveraging advanced machine learning techniques and a region-specific dataset, the study provided insights into the potential of Gated Recurrent Units (GRU) for efficient and accurate medical predictions.

1. **Use of Afrocentric Dataset:** The study utilized the SIREN dataset, which is specific to African populations. This addresses a critical gap in stroke prediction research, as most existing models are based on data from Western populations. The dataset provided culturally and regionally relevant insights, enhancing the applicability of the findings to underserved regions.
2. **Application of Gated Recurrent Units (GRU):** The use of GRU as the prediction algorithm highlighted its advantages over other machine learning models. GRU demonstrated superior performance in accuracy and computational efficiency, making it a practical tool for structured medical datasets.
3. **Feature Selection for Optimization:** The study reduced the dataset from 29 to 15 phenotypes, focusing on the most impactful features. This optimization not only improved the system’s accuracy but also reduced computational complexity, aligning with best practices in machine learning.
4. **Comprehensive Model Comparison:** By comparing the GRU-based system with traditional machine learning algorithms and Long Short-Term Memory (LSTM) networks, the study ensured a robust evaluation. The GRU model consistently outperformed these benchmarks, underscoring its effectiveness.
5. **Computational Efficiency:** The developed system achieved low prediction and evaluation times, making it highly suitable for real-time applications in healthcare settings where timely decisions are critical.
6. **Generalization and Robustness:** The system demonstrated no signs of overfitting or underfitting, as evidenced by consistent training and validation performance. This indicates the model’s reliability and potential for practical implementation.

## 6. Limitations of the study

While the study achieved notable success, certain limitations highlight areas for future improvement and research. These limitations provide context for interpreting the results and suggest directions for further exploration.

1. **Reliance on Secondary Data:** The study utilized the SIREN dataset, which, while comprehensive, may carry inherent biases or limitations from the original data collection process. Despite applying data cleaning and imputation techniques, primary data collection or alternative datasets could yield more robust results.
2. **Focus on Structured Data:** The system exclusively worked with structured data, limiting its applicability to other medical data types, such as unstructured imaging or text from electronic health records. Expanding the model to incorporate such data could enhance its utility.
3. **Lack of External Validation:** The model was tested on a single dataset, which may restrict its generalizability to other populations or regions. Validation with datasets from different contexts would provide stronger evidence of the system’s robustness.
4. **Limited Scope of Features:** Although the feature selection process identified the most impactful 15 phenotypes, it is possible that other unmeasured factors relevant to stroke prediction were not included. Future research could explore the inclusion of additional features for a more holistic analysis.
5. **Exclusion of Hybrid Models:** While GRU performed well, the study did not explore hybrid architectures, such as combining GRU with convolutional layers or attention mechanisms. Such hybrid models could potentially enhance performance further.

## 7. Conclusion

This study successfully developed a stroke prediction system using Gated Recurrent Units (GRU), achieving a high accuracy of 77.48 % with efficient prediction and evaluation times of 0.43 seconds and 0.071 seconds, respectively. By leveraging an Afrocentric dataset and employing optimized feature selection, the system demonstrated significant potential for enhancing stroke prediction in real-time healthcare applications. The GRU-based model outperformed traditional machine learning algorithms and Long Short-Term Memory (LSTM) networks, proving to be a reliable and efficient approach for structured datasets. The use of 15 carefully selected phenotypes, derived from the original 29, played a critical role in achieving these results, highlighting the importance of data-driven optimization in machine learning.

Based on the findings of this study, it is recommended that future research focus on further improving the model’s performance by integrating additional data types, such as imaging or textual data from electronic health records. Expanding the system to include hybrid architectures, such as combining GRU with convolutional or attention layers, could also enhance its predictive capabilities. Furthermore, to increase the applicability and generalizability of the system, testing and validation on diverse datasets from different regions and populations should be considered. This would ensure that the model is robust and adaptable to varying healthcare environments. Finally, implementing the system in practical healthcare settings could help evaluate its real-world performance and provide actionable insights for early stroke detection and intervention.

## Figures and Tables

**Table 1:** Requested phenotypes from the SIREN stroke database.

S/N	Name of Phenotypes	Description	Data type
1	**Formal education?**	The level of education the patients have and ranges from none to PhD	Ordinal
2	**Primary occupation?**	The source of living of the patients	Categorical
3	**Hypertension**	To show the hypertension status of the patients	Nominal
4	**Diabetes mellitus**	This shows whether a patient is DM patient or not	Nominal
5	**Cardiac disease**	This shows whether a patient is have any CVD	Nominal
6	**Family history of cvd**	This shows if any of the patient’s family had CVD	Nominal
7	**Bmi**	The value of patient’s body mass index	Numeric
8	**Alcohol usage**	The alcohol status of the patient	Nominal
9	**Blood pressure**	The value of patient’s BP	Numeric
10	**Do you have history of substance use**	This shows maybe the patient is consuming any hard drugs.	Nominal
11	**Physical activity**	This shows maybe the patients use to do any physical exercise.	Nominal
12	**Do you sprinkle salt on your food after cooking?**	This indicates maybe patients add salt to food after cooked.	Nominal
13	**Do you add salt on the table**	This indicates maybe the patients add salt to cooked food while eating.	Nominal
14	**Do you have atrial fibrillation**	This shows the AF status of the patient	Nominal
15	**Are you a vegetarian**	This shows maybe the patients eat vegies.	Nominal

**Table 2:** Summary of the SIREN Dataset.

Dataset	Data Size	No of Phenotypes	Training Size	Testing Size
SIREN	4236	15	3388	848

**Table 3:** Performance Evaluation of GRU with Different Parameters with 29 phenotypes.

S/N	Layers (Input and Hidden)	Epoch and Batch size	Average Accuracy (%)	Average Precision (%)	Average Recall (%)	Average F1Score (%)
1	64–8	50, 50	72.52	73	73	73
2	64–16	50, 16	72.88	73	73	73
3	64–16	100, 50	72.76	73	73	73
4	128–8	50, 4	71.82	70	70	70

**Table 4:** Performance Evaluation of GRU with Different parameters with 15 phenotypes.

S/N	Layers (Input and Hidden)	Epoch and Batch size	Avg. Accuracy (%)	Avg. Precision (%)	Avg. Recall (%)	Avg. F1Score (%)	Prediction Time (Sec)
1	64–8	50, 50	72.64	73.00	73.00	73.00	
2	128–8	100,64	75.00	75.00	75.00	75.00	0.82
3	128–8	120,32	74.41	74.00	74.00	74.00	2.18
4	128–8	100, 32	76.65	77.00	77.00	77.00	0.43
5	128–8	150,8	77.48	77.00	77.00	77.00	0.42

**Table 5:** Evaluation Result of the developed system.

S/N	EVALUATION METRICS	RESULT
1	Average Accuracy (%)	77.48
2	Average F1Score (%)	77.00
3	Area Under Curve (AUC)	0.85
4	Mean Absolute Error (MAE)	0.31
5	Prediction time (secs)	0.42
6	Evaluation Time (sec)	0.071

**Table 6:** Comparison of LSTM and GRU Evaluation Metrics.

S/N	LSTM layers	GRU layers	Epochs and Batch-size	LSTM Avg. Accuracy %	GRU Avg. accuracy %	LSTM Prediction time	GRU prediction time
1	128–16	128–8	100, 8	74.88	77.48	2.23	0.43
2	64–16	64–8	50, 50	66.27	72.29		
2	100–16	128–8	100,64	63.09	76.65	0.68	0.43

**Table 7:** Comparison of the Developed systems with some Machine Learning Algorithms.

S/N	Algorithm	Average Accuracy (%)	Average Precision (%)	Average Recall (%)	Average F1 score (%)
1	Logistic Regression	73.58	74	74	74
2	Support Vector Machine	72.88	73	73	73
3	K-Nearest Neighbor (k=3)	71.82	72	72	72
4	Developed Stroke	77.48	77	77	77
